# Neurodevelopmental Outcomes in Preterm Infants Receiving a Multicomponent vs. a Soybean-Based Lipid Emulsion: 24 Month Follow-Up of a Randomized Controlled Trial

**DOI:** 10.3390/nu15010058

**Published:** 2022-12-23

**Authors:** Francesca Gallini, Maria Sofia Pelosi, Domenico Umberto De Rose, Maria Coppola, Simonetta Costa, Domenico Marco Romeo, Carmen Cocca, Luca Maggio, Francesco Cota, Alessandra Piersanti, Daniela Ricci, Giovanni Vento

**Affiliations:** 1 Neonatology Unit, Department of Woman and Child Health and Public Health, Fondazione Policlinico Universitario “Agostino Gemelli” IRCCS, 00168 Rome, Italy; 2 Dipartimento di Scienze della Vita e Sanità Pubblica, Facoltà di Medicina e Chirurgia, Università Cattolica del Sacro Cuore, 00168 Rome, Italy; 3 Neonatal Intensive Care Unit, Medical and Surgical Department of Fetus-Newborn-Infant, “Bambino Gesù” Children’s Hospital IRCCS, 00165 Rome, Italy; 4 Pediatric Neurology Unit, Department of Woman and Child Health and Public Health, Fondazione Policlinico Universitario “Agostino Gemelli” IRCCS, 00168 Rome, Italy; 5 Neonatology Unit, San Camillo Forlanini Hospital, 00152 Rome, Italy; 6 National Centre of Services and Research for the Prevention of Blindness and Rehabilitation of Low Vision Patients-International Agency for the Prevention of Blindness (IAPB) Italia Onlus, 00185 Rome, Italy

**Keywords:** multicomponent lipid emulsion, parenteral nutrition, SMOFlipid, Intralipid, very-low-birth-weight infants, preterm infants, Griffith’s Mental Development Scales, neonatal outcome

## Abstract

Background: Few studies in the literature have analyzed the long-term neurodevelopmental outcomes of the administration of a multicomponent versus a soybean-based lipid emulsion (LE) in preterm infants receiving parenteral nutrition (PN). A recent randomized controlled trial conducted in our unit provided evidence of better growth in head circumference during the hospital stay in those who received a multicomponent LE. Methods: This is a 24 month follow-up study of preterm infants, previously enrolled in a randomized trial, who received a multicomponent LE (SMOFlipid^®^) or a standard soybean-based one (Intralipid^®^). We evaluated neurodevelopmental outcomes at 24 months of corrected age (CA) in the two groups. Results: Ninety-three children were followed up to the age of 24 months CA. Due to the peculiar time frame of the SARS-CoV-2 pandemic, neurodevelopmental outcomes were evaluated only in 77 children: 37 in the SMOFlipid^®^ group and 40 in the Intralipid^®^ group. No differences in major disability rates or in Griffith’s evaluation were found between the two groups. Conclusions: In our population study, the administration of a multicomponent LE containing fish oil, compared to a soybean-based LE, had no significant effects on neurodevelopmental outcomes in preterm infants at 24 months CA.

## 1. Introduction

Preterm birth complications are still the leading cause of mortality in children under the age of 5 years worldwide [1]. Furthermore, beyond mortality, extremely preterm infants have an increased risk of impaired neurodevelopment compared to their term-born counterparts. A possible strategy to lessen the negative effects of preterm birth on neurodevelopment is to optimize nutrition [2], reducing complications of extrauterine growth restriction [3]. In very preterm neonates, the advantages of starting parenteral nutrition (PN) on the first days of life appear to exceed the risks of infections [4].

An important role is played by ω-3 long-chain polyunsaturated fatty acids (LC-PUFAs), docosahexaenoic acid (DHA), and eicosapentaenoic acid (EPA), which are essential for the adequate structural and functional development of the brain and retina of the fetus [5,6,7].

Preterm newborns have been given intravenous fats made from a pure soybean-based lipid emulsion (SLE) that is high in ω-6 polyunsaturated fatty acids (PUFAs) and low in ω-3 PUFAs, for a long period. Newer lipid emulsions (LEs) have been introduced in the last years to provide a better balance of lipids supply from various sources; these multicomponent lipid emulsions (MLEs) contain olive oil, fish oil, and medium-chain triacylglycerols (MCTs) oil in addition to soybean oil [7].

Our group recently published the results of a randomized controlled trial (RCT) designed to assess the efficacy of an MLE (SMOFlipid^®^ 20%, Fresenius Kabi, Italy) on the growth of head circumference (HC), providing evidence that an MLE is associated with improved HC growth at 36 weeks postmenstrual age (PMA) or at discharge in comparison with a pure SLE (Intralipid^®^ 20%, Fresenius Kabi, Italy) [8]. The aim of the present study was to analyze the effect of this MLE on the neurodevelopmental outcomes of study participants at 24 months of corrected age (CA).

## 2. Materials and Methods

### 2.1. Study Design

The protocol of this study was approved by the Ethics Committee of the “Fondazione Policlinico Universitario Agostino Gemelli—IRCCS” (Rome, Italy), and written informed consent was obtained from the parents in all cases.

This is a 24 month follow-up of infants enrolled in the neonatal “SMOFlipid^®^ vs. Intralipid^®^” RCT [8], discharged from the Neonatal Intensive Care Unit (NICU) of “Fondazione Policlinico Universitario Agostino Gemelli—IRCCS” Hospital in Rome (Italy). Enrolled patients were born between 2016 and 2019, including newborns with gestational age (GA) ≤30 weeks and/or a birth weight ≤1250 g who received PN. Infants were randomized to receive, from the moment they started PN in the NICU within 24 h of life, a multicomponent lipid emulsion (SMOFlipid^®^ 20%, Fresenius Kabi, Germany; study group) or the traditional soybean oil-based lipid emulsion (Intralipid^®^ 20%, Fresenius Kabi, Germany; control group). Neonates with congenital malformations or chromosomal syndromes and those who developed post-hemorrhagic hydrocephalus were excluded. After NICU discharge, all infants were enrolled in a 2 year anthropometric and neurodevelopmental follow-up program.

The primary outcome was the evaluation of long-term neurodevelopmental outcomes at 24 months CA. The secondary outcomes were short-term neurodevelopmental outcomes (6 and 12 months of CA) and auxological parameters at different timepoints (40 weeks postmenstrual age—PMA, 6, 12, and 24 months of CA).

### 2.2. Follow-Up and Neurodevelopmental Outcomes

Clinical follow-up visits were scheduled at 40 weeks of postmenstrual age (PMA) and at 1, 3, 6, 9, 12, 18, and 24 months of CA, according to our Neonatal High-Risk Follow-up Program, with a standardized growth and neurodevelopmental assessment [9]. At each visit, the anthropometric evaluation was performed by assessing the infant’s weight using electronic scales, while the determination of the length was carried out by using the Harpenden neonatometer or a fixed stadiometer, evaluating the crown-heel measurement. Head circumference was determined by measuring the maximal occipital–frontal circumference through a centimeter tape of inextensible material. These values were converted to the sex- and age-specific z-score of weight, height, and head circumference at 12 and 24 months of CA, according to CDC 2000 growth charts [10].

The short-term neurodevelopmental outcome was evaluated at 6 and 12 months of CA by an expert pediatric neurologist using the Hammersmith Infant Neurological Examination (HINE). The HINE includes 26 items, assessing five different aspects: cranial nerve function, posture, movements, tone, and reflexes. For each item, a different score can be obtained (from 0 to 3), where 3 is the optimal score, and the maximum total global score (GS) is 78. At 6 months of CA, preterm-born infants with a GS equal to or above 70 were considered with an optimal score, whereas, at 12 months of CA, a GS of at least 73 is considered optimal [11].

The long-term neurodevelopmental outcome was evaluated at 24 months of CA by an expert pediatric neurologist using Griffith’s Mental Developmental Scales (GMDS) (0–2 years) [12]. The Italian-validated translation of the administration manual was used, as previously described [13]. GMDS yields five subscales: locomotor, personal–social, hearing and speech, eye and hand coordination, and performance. The subscales give standardized scores for each domain and a composite developmental quotient. Cognitive outcome was classified as normal when Griffith’s developmental quotient (GDQ) was >85, borderline when GDQ was from 70 to 85, and delayed when GDQ was lower than 70 [14]. At 24 months of CA, we defined “major disability” as the presence of at least one among the following: cerebral palsy (CP) according to Bax classification [15], cognitive impairment defined by a GDQ < 70, visual impairment (visual acuity <6/60 in the better eye) [16], and hearing impairment (as deafness requiring bilateral hearing aids or unilateral/bilateral cochlear implants) [17].

### 2.3. Statistical Analysis

All data were collected prospectively and stored on a dedicated database. Data are presented as numbers and percentages for categorical variables. Continuous variables are expressed as the mean ± standard deviation (SD) if they were normally distributed or as median and interquartile range if normality could not be accepted. Comparisons between continuous variables were performed using the Student’s *t*-test in the case of normal distribution and the Mann–Whitney U-test in the case of non-normal distribution. Categorical variables were compared using Fisher’s exact test or chi-square with Yates correction. A two-tailed *p*-value < 0.05 was considered significant. Statistical analysis was performed using software programs Microsoft Excel (2016 for Windows, Redmond, Washington, DC, USA) and SPSS (version 25.0 for Windows, Armonk, NY, US) to analyze the effect of this MLE on the neurodevelopmental outcomes of study participants at 24 months of corrected age (CA).

## 3. Results

### 3.1. Study Population

The study population included 101 preterm infants who were discharged from our NICU after participating in a previous RCT [8]. Fifty-one children received MLE (SMOFlipid^®^), while 50 received SLE (Intralipid^®^). There were no significant differences in the time to reach full enteral feeding (35.8 ± 21.4 days in the MLE group versus 37.2 ± 28.6 days in the SLE group, *p* = 0.780) and in the duration of parenteral nutrition (39.7 ± 31.4 days in the MLE group versus 33.4 ± 28.4 days in the SLE group, *p* = 0.293).

**Figure 1:**
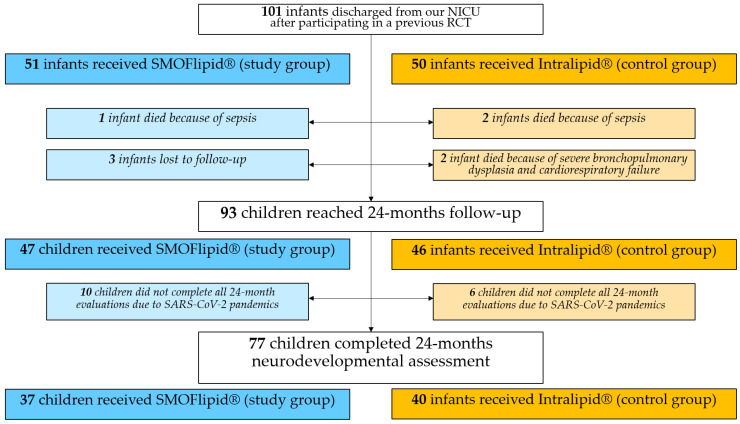
Population and design of the study.

Figure 1 shows the quantitative allocation of the infants between the two study arms and, within each group, how many children reached the final follow-up milestone. Of the 101 infants included, five died after discharge (one belonging to the study group and four belonging to the control group). Long-term outcomes were evaluated in a final population of 93 infants: 47 enrolled in the study group and 46 in the control group.

There were no differences between the two groups in terms of neonatal characteristics (Table 1). During the NICU stay, no significant differences in the prevalence of culture-proven sepsis, patent ductus arteriosus (PDA) requiring treatment, retinopathy of prematurity (ROP) requiring treatment, severe intraventricular haemorrhage (IVH), periventricular leukomalacia (PVL), and bronchopulmonary dysplasia (BPD) that could have influenced short-term and long-term outcomes were observed between the two groups. However, a higher prevalence of surgical necrotizing enterocolitis (NEC) and a longer length of stay in the hospital were observed in the study group without reaching a statistically significant difference.

### 3.2. Auxological Outcomes

We found no long-term growth differences between the two groups at 12 and 24 months of CA (Table 2 and Table 3, respectively).

### 3.3. Short-Term Neurodevelopmental Outcomes

**Figure 2:**
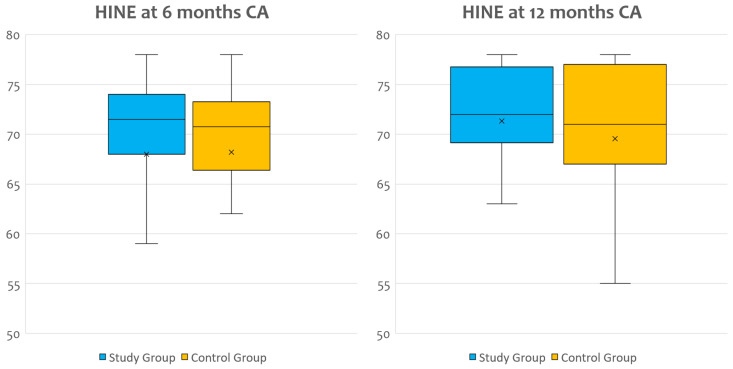
Global score in the two groups at Hammersmith Infant Neurological Examination (HINE) at 6 and 12 months CA.

At HINE evaluation, median values of GS resulted within the normal range in both groups of infants, and they were comparable without reaching a statistically significant difference at either 6 or 12 months CA (Figure 2).

### 3.4. Long-Term Neurodevelopmental Outcomes

Due to the peculiar timeframe of the SARS-CoV-2 pandemic, neurodevelopmental outcomes at 24 months CA were not evaluated for all infants. In fact, a total of 77 children were assessed, 37 out of 47 children of the SMOFlipid group (79%) and 40 out of 46 children of the Intralipid group (87%). Among the infants who were assessed at 24 months, no differences in major disability rates were found between the two groups (Table 4).

Concerning GMDS evaluation at 24 months of CA, the distribution of normal, borderline, and delayed GDQ was similar in the two groups (Table 5). We found no differences in GDQ or in Griffith’s subscales between the two groups (Table 6).

## 4. Discussion

In this prospective study, we evaluated the follow-up of infants enrolled in a previous RCT who were randomized to receive a multicomponent lipid emulsion (SMOFlipid^®^ 20%, Fresenius Kabi, Germany; study group) or the traditional soybean oil-based lipid emulsion (Intralipid^®^ 20%, Fresenius Kabi, Germany; control group). In our cohort, the use of an LE containing fish oil in preterm-born children did not result in improved neurodevelopmental outcomes at 24 months CA, contrary to our prediction, according to the better trend in the growth of head circumference initially observed during NICU stay. Long-term neurodevelopmental outcomes seemed not to be affected in terms of overall disability, either evaluated as the prevalence of infants with major disabilities or as the prevalence of infants with normal, borderline, and pathologic developmental quotient (GDQ).

Our data are quite interesting, considering that most studies about the use of SMOFlipid^®^ were related only to short-term neonatal outcomes [8,18,19,20]. Furthermore, only one study by Thanhaeuser et al. previously analyzed the outcomes of preterm infants receiving MLE or SLE after randomization. Similarly, they reported that it did not improve the neurodevelopment of extremely-low-birth-weight infants, assessed using Bayley-III at 12 and 24 months CA [21].

Results from retrospective studies are controversial. Biagetti et al. described that they found no differences in motor and language scores and in the incidence of neurodevelopmental disabilities between infants who received routine PN with different LEs, with and without fish oil [22]. Conversely, Torgalkar et al. noted lower odds of adverse neurodevelopmental outcomes in preterm infants who received SMOFlipid^®^ rather than in those who were given Intralipid^®^ after a pre–post comparative cohort study without any randomization [23]. In addition, Chen et al. evidenced a decreased incidence of epilepsy, cerebral palsy, developmental disorder, and attention-deficit hyperactivity disorder (ADHD) at 2 years among children who previously received LEs with fish oil rather than a standard soybean oil-based emulsion [24]. However, these results are difficult to compare with ours since Chen et al. conducted a retrospective cross-sectional study and not an RCT; furthermore, the mean gestational age of the infants enrolled in their study was higher (mean of 32.2 weeks GA) than that of our infants (mean of 28.1 weeks GA), and because the parenteral lipid emulsions under analysis were different (Lipofundin^®^ instead of Intralipid^®^). Of note, Chen’s study is the only one available in the literature that evaluated the neurodevelopmental outcomes of preterm infants at 5 years of age.

The biological plausibility of better neurodevelopment in children who previously received an LE containing fish oil is related to the different ratios of available fatty acids. The issue is that pure soybean oil-based emulsions contain high levels of ω-6, which can increase proinflammatory eicosanoid biosynthesis leading to increased oxidative stress and low levels of ω-3 (DHA and EPA) [25], instead of more balanced multicomponent lipid emulsions. Furthermore, the better growth of head circumference, which we previously demonstrated in the initial RCT, was also demonstrated by Vlaardingerbroek et al. in another RCT in 2013 [18]. Moreover, the growth of head circumference is considered to be a good tool for predicting neurodevelopmental outcomes [3,26].

Our study presented some limitations. The major limitation was the power of the study; we could have failed to find statistically significant differences between the two parenteral LEs in any of the analyzed long-term outcomes because the initial study was powered on short-term outcomes and not on long-term ones. Moreover, some children did not complete all neurodevelopmental evaluations because of the frameshift of the SARS-CoV-2 pandemic, further reducing the number of infants studied in the original RCT.

The major strength of our study was the design of the study (follow-up of an RCT, with data derived from a randomized population, thus eliminating the possibility of confounding factors). Moreover, we analyzed the long-term follow-up data of very small infants, all with gestational age (GA) ≤30 weeks and/or a birth weight ≤1250 g.

Therefore, with the purpose of consistently evaluating the long-term neurodevelopmental outcomes of infants administered with SMOFlipid^®^ parenteral lipid emulsion, additional prospective multicentric studies involving more infants are strongly suggested. Neurovisual assessments to evaluate more specific outcomes, such as visual function ones and neuroimaging through diffusion-weighted magnetic resonance imaging (DWI MRI) tractography to study the optical tracts, may be very useful. Then, a long-term assessment until school age should be planned to identify and monitor possible neurosensory and intellectual sequelae, as well as behavioral disturbances, that could compromise academic performance.

## 5. Conclusions

Beyond the transient enhanced growth of head circumference observed in early life, we failed to find improved neurodevelopmental outcomes at 24 months CA in preterm infants who previously received a multicomponent lipid emulsion containing fish oil (SMOFlipid^®^) during NICU stay, compared to those who were given pure soybean oil-based emulsion (Intralipid^®^). In addition, due to the similar auxological outcomes that we reported, we demonstrated the long-term safety of this multicomponent lipid emulsion.

Further multicenter randomized controlled trials are needed to confirm our findings, including a larger sample of very preterm infants and focusing on more specific outcomes.

## Figures and Tables

**Table 1:** Neonatal characteristics in the two groups.

	Study Group (n = 47)	Control Group (n = 46)	*p*-Value
Males, n (%)	22 (47)	18 (39)	0.29
Gestational age, weeks	28.0 ± 2.5	28.2 ± 1.9	0.80
Birth weight, g	865 ± 225	895 ± 220	0.61
Birth weight Z-score, SD	−0.78 ± 1.07	−0.68 ± 1.03	0.61
Birth length, centimeters	34.3 ± 3.2	34.8 ± 3.5	0.56
Birth length Z-score, SD	−0.82 ± 1.07	−0.63 ± 1.36	0.47
Birth head circumference, Centimeters	24.3 ± 2.3	24.6 ± 2.3	0.68
Birth head circumference Z-score, SD	−0.93 ± 0.89	−0.82 ± 1.01	0.51
Small for gestational age, n	19 (40)	13 (28)	0.22
Prenatal steroids, n (%)	39 (83)	37 (80)	0.75
Postnatal steroids, n (%)	7 (15)	5 (11)	0.56
Culture-proven sepsis, n (%)	14 (30)	11 (24)	0.57
NEC stage >2A, n (%)	3 (6)	0	0.24
PDA requiring treatment, n (%)	21 (45)	20 (43)	0.98
BPD, n (%)	22 (47)	18 (39)	0.46
ROP requiring treatment, n (%)	9 (19)	9 (20)	0.92
IVH stage ≥2, n (%)	0	0	1.00
PVL stage ≥2, n (%)	0	0	1.00
Length of stay, days	77 (48–113)	63.5 (48–93.8)	0.31

**Table 2:** Anthropometric evaluation in the two groups at 12 months CA.

	Study Group (n = 47)	Control Group (n = 46)	*p*-Value
Corrected age at evaluation, months	12.2 ± 0.6	12.5 ± 1.1	0.12
12 month weight, g	8627 ± 1326	8792 ± 1140	0.52
12 month weight Z-score, SD	−1.26 ± 1.47	−1.00 ± 1.20	0.35
12 month length, centimeters	73.1 ± 4.1	73.8 ± 3.0	0.33
12 month length Z-score, SD	−0.63 ± 1.35	−0.32 ± 1.03	0.22
12 month head circumference, centimeters	44.9 ± 2.1	45.2 ± 1.6	0.33
12 month head circumference Z-score, SD	−0.71 ± 1.54	−0.25 ± 1.25	0.12

**Table 3:** Anthropometric evaluation in the two groups at 24 months CA.

	Study Group (n = 47)	Control Group (n = 46)	*p*-Value
Corrected age at evaluation, months	23.9 ± 1.1	24.0 ± 1.2	0.48
24 month weight, grams	10553 ± 1448	11070 ± 1632	0.25
24 month weight Z-score, SD	−1.61 ± 1.43	−1.18 ± 1.32	0.14
24 month length, centimeters	84.1 ± 4.3	84.9 ± 3.9	0.35
24 month length Z-score, SD	−0.65 ± 1.18	−0.36 ± 0.97	0.20
24 month head circumference, centimeters	46.9 ± 2.0	47.3 ± 1.7	0.25
24 month head circumference Z-score, SD	−0.59 ± 1.24	−0.45 ± 1.16	0.17

**Table 4:** Long-term neurodevelopmental outcomes in the two groups at 24 months CA.

	Study Group (n = 37)	Control Group (n = 40)	*p*-Value
No major disability, n (%)	32 (86)	34 (85)	1.00
Major disability, n (%)	5 (14)	6 (15)	1.00
Cerebral palsy, n (%)	1 (2.7)	2 (5)	1.00
Cognitive impairment, n (%)	4 (10.8)	4 (10)	1.00
Visual impairment, n (%)	1 (2.7)	1 (2.5)	1.00
Hearing impairment, n (%)	2 (5.4)	1 (2.5)	0.61

**Table 5:** Distribution of Griffith’s Developmental Quotient (GDQ) in the two groups at 24 months CA.

	Study Group (n = 37)	Control Group (n = 40)	*p*-Value
Normal GDQ, n (%)	28 (76)	33 (83)	0.46
Borderline GDQ, n (%)	5 (13)	3 (7)	0.47
Cognitive impairment, n (%)	4 (11)	4 (10)	1.00

**Table 6:** Total Griffith’s Developmental Quotient (GDQ) and Griffith’s subscale scores in the two groups at 24 months of corrected age.

	Study Group (n = 34)	Control Group (n = 36)	*p*-Value
(A) Locomotor	106 ± 27	101 ± 25	0.44
(B) Personal-Social	114 ± 28	111 ± 31	0.70
(C) Hearing and Language	90 ± 26	90 ± 23	0.98
(D) Eye and Hand Coordination	100 ± 16	102 ± 17	0.72
(E) Performance	118 ± 27	117 ± 28	0.81
Total GDQ score	106 ± 20	104 ± 19	0.75

## Data Availability

The original contributions presented in this study are included in the article; further inquiries can be directed to the corresponding author.
